# RNA-Based Strategies for Cell Reprogramming toward Pluripotency

**DOI:** 10.3390/pharmaceutics14020317

**Published:** 2022-01-28

**Authors:** Anaëlle Bailly, Ollivier Milhavet, Jean-Marc Lemaitre

**Affiliations:** 1 IRMB, University Montpellier, INSERM, 34295 Montpellier, France; 2 INGRAALYS, SA, IRMB, Incubator Cyborg, 34295 Montpellier, France; 3 IRMB, University Montpellier, INSERM, CNRS, 34295 Montpellier, France; 4 SAFE-iPSC Facility, CHU Montpellier, 34295 Montpellier, France

**Keywords:** aging, senescence, RNA, epigenetics, stem cells, reprogramming, iPSC

## Abstract

Cell therapy approaches to treat a wide range of pathologies have greatly benefited from cell reprogramming techniques that allow the conversion of a somatic cell into a pluripotent cell. Many technological developments have been made since the initial major discovery of this biological process. Recently reprogramming methods based on the use of RNA have emerged and seem very promising. Thus, in this review we will focus on presenting the interest of such methods for cell reprogramming but also how these RNA-based strategies can be extended to eventually lead to medical applications to improve healthspan and longevity.

## 1. Introduction

Cell therapy approaches are particularly suitable for treating diseased or aging tissues or organs. They also allow the restoration of deficient or absent cellular or tissue functions. The use of stem cells, and, in particular, pluripotent stem cells with their differentiation properties, has made it possible to substantially expand the field of clinical intervention opportunities. However, it is with the discovery of cellular reprogramming that the stem cell thematic has really taken off. Cellular reprogramming changes the identity of a somatic cell into a pluripotent cell with the same cellular characteristics—in particular, self-renewal and differentiation—as an embryonic stem cell. Cellular reprogramming induces a global remodeling of epigenetic marks to reset the epigenetic landscape and, thus, to change the cell identity and revert it to a pluripotent embryonic-like state. Homologous transplantation strategies can then be established, since the reprogramming of a somatic cell from a specific donor into pluripotent cells can be performed before reinjection, after redifferentiation into cells of interest, of the same patient. The reprogrammed cells can also be modified or corrected before redifferentiation to produce the cells, tissues, or organs to be replaced. Pluripotent cells from multiple sources can be banked before being used in a compatible patient in the coming of age of off-the-shelf cell therapies.

Multiple obstacles remain, and constraints need to be addressed before concrete clinical applications. Among them, producing reprogrammed cells remains an awkward but critical step. The use of strategies based on mRNA to express the factors needed for cell reprogramming has rapidly emerged as a promising technology to achieve this goal. Hence, in this review, after a brief revisiting of the state-of-the-art various technologies, we will focus on methods based on RNA that induce the conversion of somatic cells into pluripotent cells. We will frame these technological advances in the context of recent cutting-edge approaches to reverse age-related cell and tissue phenotypes by reprogramming them towards pluripotency.

## 2. Reprogramming

### 2.1. History

In 1962, John B. Gurdon laid the groundwork for reprogramming when he demonstrated that the nucleus of a somatic cell, transferred into an enucleated egg, was able to return to a state of pluripotency and generate a tadpole [1]. Later, using the same principle, Ian Wilmut and colleagues were the first to clone a mammal, “Dolly” the sheep [2]. This technology, called somatic cell nucleus transfer (SCNT), proves that the nucleus of a somatic cell contains all the genetic information needed to revert to a state of pluripotency and that the egg contains all the factors necessary for regulating the gene expression profile of each cell.

In 2001, another reprogramming strategy was developed, called cell fusion. Indeed, Tada et al. obtained mouse cells expressing pluripotency genes by fusing somatic cells with embryonic stem cells (ESCs), implying that ESCs also contain reprogramming factors [3].

Altogether, these findings paved the way for the emergence of the knowledge required to manipulate the program of somatic cells towards pluripotency at the start of the 21st century. To achieve this goal, Shinya Yamanaka and his team screened 24 transcription factors, known to be associated with the mouse embryonic state, by overexpressing them in fibroblasts cultured in vitro. Through a process of selection by elimination, in 2006, they zeroed in on a cocktail of four factors: *Oct4*, *Sox2*, *Klf4* and *c-Myc* (OSKM), named “the Yamanaka’s factors” or “the Yamanaka’s cocktail”, allowing the reprogramming of murine fibroblasts into cells with properties similar to ESCs [4]. These cells were called induced pluripotent stem cells (iPSCs), and in 2007, the experiment was successfully reproduced with human fibroblasts [5].

These iPSCs have all the characteristics of bona fide pluripotent stem cells; they can self-renew and differentiate into cells from the three embryonic layers (ectoderm, mesoderm and endoderm). The discovery of induction to pluripotency means we can now produce patient-specific and/or disease-specific iPSCs as a promising alternative to ESCs for regenerative medicine, drug discovery and disease modeling. iPSCs are a potentially unlimited source of cells with infinitely less ethical problems than ESCs. It should be stressed that, in theory, a single iPSC colony is sufficient to replenish all the cells of a given organism, and this opens tremendous clinical opportunities. The 2012 Nobel Prize in Physiology and Medicine was awarded to both Yamanaka and Gurdon for breaking through the reversibility of cell differentiation [6].

### 2.2. Somatic Cell Sources

**Figure 1:**
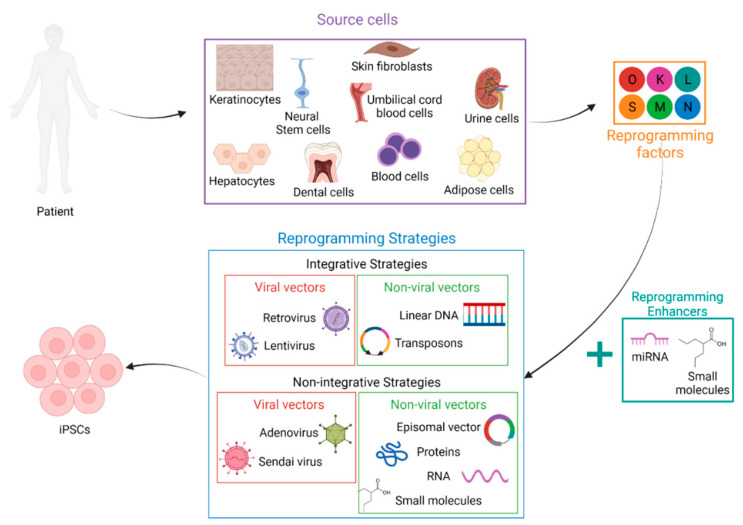
The generation of iPSC lines requires strategic choices adapted to the clinical context and objectives. The main steps include the selection of the starting cell type, the choice of the combination of reprogramming factors, the use (or not) of reprogramming enhancers and, finally, deciding upon a reprogramming strategy. Created with BioRender.com (accessed on 30 November 2021).

Since the first generation of iPSCs, researchers have endlessly improved and fine-tuned this technology and pursued the development of new reprogramming strategies (Figure 1). Among the different axes, the cell types used as the starting material were particularly explored. Indeed, ideally, cells to reprogram should be easily collectable with a minimally invasive procedure. They should also be easily cultivable and sufficiently proliferative to obtain many cells free from critical somatic mutations and chromosomal aberrations. Finally, the harvested cells must be capable of generating iPSCs with high efficiency.

To date, fibroblasts are the most widely used source of cells for reprogramming [5,7,8,9]. Skin biopsy is actually a very well-mastered technique, and the fibroblast culture protocols are well-established. Additionally obtained from skin biopsies, melanocytes can be reprogrammed [10]. More easily available, although their culture can be complicated, peripheral blood cells are also widely used: CD34^+^ hematopoietic stem cells [11,12,13], blood mononuclear cells (MNCs) [14] and T-lymphocytes [11,15,16,17]. The starting cell types can also be obtained from surgical and biological waste. This is, for example, the case with mesenchymal stem cells derived from adipose tissue [18,19,20], mesenchymal stem cells derived from dental tissue [21], cells derived from umbilical cord blood [22,23,24,25] or cells derived from urine [26,27,28]. However, as these sources are mostly obtained from sporadic invasive surgery, their procurement is not routine, and they are not really suitable for clinical autologous grafting. Neural stem cells (NSCs) [29] and hepatocytes [30] can also be cited as sources of iPSCs. Finally keratinocytes are more reprogrammable than fibroblasts and much more available [31,32,33].

### 2.3. Reprogramming Factors

After selecting the cells to reprogram, a suitable cocktail of factors must be chosen. While the original Yamanaka OCT4, SOX2, KLF4 and C-MYC (OSKM) cocktail has been widely used on a large number of cell types and with a large number of vectors [11,12,17,18,21,26,31,34,35,36], other combinations have also been suggested. In 2007, Yu et al. proposed the OSNL cocktail composed of two common factors and two different ones, i.e., OCT4, SOX2, NANOG and LIN28 [7].

Since then, several variations in the choice of these factors have been proposed to improve the efficiency of reprogramming or to overcome barriers. We can, for example, quote the studies of Liao et al. [37] and our study by Lapasset et al. [38] using a six-factor cocktail, OSKMNL. Liao et al. revealed that the reprogramming efficiency of fibroblasts with these six factors is 10 times higher than that obtained with OSNL, while we demonstrated that the combination of the six factors makes it possible to reprogram senescent cells and the oldest cells from centenarian donors into iPSCs. Senescence was previously described as a barrier to reprogramming [39,40,41,42,43,44]. That study therefore opened up new horizons for countering the adverse effects of aging.

The choice of how many and which reprogramming factors to use also depends on the starting cell type. Indeed, studies have proven that the number of reprogramming factors can be reduced by taking advantage of the endogenous expression of certain factors by the selected cell type. For instance, it has been shown that SOX2 is not necessary to reprogram melanocytes [10], that umbilical cord blood stem cells can be reprogrammed with only OCT4 and SOX2 [22,23] and even that OCT4 was sufficient to reprogram human neural stem cells [29]. Recently, murine fibroblasts have even been reprogrammed without OCT4, which was considered essential at the time [45].

### 2.4. Reprogramming Enhancers

Since many epigenetic modifications occur during the reprogramming process and chromatin marks are fundamental for iPSC generation, chemical compounds and small molecules modifying epigenetic processes are studied for their impact on reprogramming. It has thus been demonstrated that the reprogramming efficiency is enhanced by inhibitors of DNA methyltransferase such as 5-azacytidine (AZA) [46], inhibitors of histone deacetylase such as valproic acid [47] or sodium butyrate [48] or inhibitors of histone demethylase such as parnate [49]. Indeed, a combination of parnate and the glycogen synthase kinase-3 inhibitor CHIR99021 allowed iPSC generation from human keratinocytes with just two factors: OCT4 and KLF4 [49]. Similarly, Huangfu et al. demonstrated that valproic acid could replace the oncogene C-MYC or KLF4 for reprogramming primary human fibroblasts with only two factors: OCT4 and SOX2 [47]. In addition, the combination of valproic acid and vitamin C, which induces the demethylation of DNA and alleviates cell senescence, increased the number of iPSC colonies obtained after reprogramming [50]. Sodium butyrate also increased the reprogramming efficiency of fetal or adult human fibroblasts by 15–51 times [48]. Finally, late treatment with AZA facilitates the transition to the pluripotent state of cells and thus improves the reprogramming efficiency [46]. These small molecules have the considerable advantage of being able to penetrate the cytoplasm of the cell, and they minimize the risk of mutation because they can be removed at any time during reprogramming.

MicroRNAs (miRNAs) have also been shown to play a crucial role in reprogramming and to improve the reprogramming efficiency in many cases. The implication and interest of miRNAs will be covered in the second part of this review.

### 2.5. Reprogramming Strategies

“Delivery strategies” refers to the methods that introduce the reprogramming factors into cells, and these strategies have been under scrutiny, as they are a key parameter for clinical applications due to their major impact on efficacy and safety. Many methods have been evaluated, and they can be classified into two categories: integrative and nonintegrative systems, where the former involves integration of the genetic material of the vector into the host’s genome. Among the integrative systems, viral vectors (retrovirus and lentivirus) can be distinguished from nonviral vectors (linear DNA and transposons). Likewise, nonintegrating viral vectors (the adenovirus and the Sendai virus) and nonintegrating nonviral vectors (episomal vectors, proteins, chemical molecules and RNA) can be discerned.

#### 2.5.1. Integrative Strategies

Retrovirus/lentivirus

Retroviruses were the first delivery strategy for the generation of iPSCs [4,5], and they have been widely used for several years now [31]. These vectors have a cloning capacity of 6–8 kb and a very high transduction efficiency. However, efficient transduction requires actively dividing cells, and therefore, this method is not suitable for reprogramming nondividing or poorly dividing cells, and this is therefore a great limitation of this strategy. Rapidly, retroviruses were replaced by lentiviruses. Lentiviruses have been used to integrate into and reprogram nondividing cells [7], allowing the reprogramming of most cell types. Lentiviruses have a high reprogramming efficiency and are easy to generate. A challenge for retroviruses and lentiviruses is the potential lack of control of silencing of the expression of the integrated transgene, preventing either the acquisition of a cell autonomous maintenance of pluripotency or impacting differentiation by a non-fully silenced expression of transgenes or their re-expression during the differentiation process, potentially leading to tumor cells. [51]. The risk of tumorigenesis is also increased, because viral transgenes are inserted into multiple and random sites in the genome of the iPSCs.

To limit this risk, improvements to the system have been proposed by using either Doxycycline inducible transgenes [52,53,54] or the excisable Cre-loxP system, which theoretically generates iPSCs free of transgenes [55], or by the use of nonintegrating lentiviral viruses (NILVs, defective in integrase activity) but with a 50-fold lower efficiency than their integrating counterpart [56].

Transposons

The PiggyBac transposon [57,58,59] and the Sleeping Beauty system [35,60] have been developed as other integrative delivery strategies with a decreased risk of mutation but with a lower reprogramming efficiency than that of the viral vectors.

Transfection of linear DNA

To avoid methods based on viral vectors, linear DNA was also used by transfection into cells with liposomes or electroporation to reprogram cells into iPSCs with a polycistronic vector, expressing all the cDNAs from a single promoter. In 2009, Kaji et al. use a nonviral polycistronic vector to reprogram murine fibroblasts, with a Cre-loxP recombination system to excise the reprogramming factors [57]. Although attractive for its simplicity, this system has very low efficiency, notably due to the low transfection rate.

#### 2.5.2. Nonintegrative Strategies

Adenovirus

The first integration-free reprogramming was based on an adenoviral system, developed in 2008 by Stadtfeld et al. to infect murine hepatocytes with a replication-incompetent adenovirus expressing the OSKM factors [36]. In 2009, Zhou and Freed also reported iPSC generation from human fibroblasts with an adenovirus expressing the original four reprogramming factors [61].

Although not integrative, the use of adenoviruses as a delivery strategy for reprogramming is relatively inefficient because of their low infection efficiency and their rapid elimination from proliferative host cells.

Sendai virus

The Sendai virus is an enveloped, single-stranded, negative-sense RNA virus that replicates directly in the cytoplasm of the host cell, without any DNA intermediary. These characteristics explain its widespread use for reprogramming a large number of cell types: fibroblasts [62], T-lymphocytes [16], peripheral blood mononuclear cells [63], mesenchymal stem cells derived from dental tissue [64] or even keratinocytes [65].

The Sendai virus offers many benefits. First, it enables rapid and strong protein expression associated with high transduction levels, providing rapid reprogramming kinetics. In addition, it has a high reprogramming efficiency on many cell types. Finally, the lack of a DNA phase leads to the impossibility of host genome integration and genetic silencing by epigenetic modifications. The advantage of long-term expression may raise an issue when the endogenous expression of pluripotency is activated, because for therapeutic purposes, the expression of the factors must be strictly controlled, and thus, the lack of expression from the virus, as well as its presence, have to be monitored. Accordingly, to ensure transgene removal, temperature-sensitive vectors allowing the elimination of the virus at a temperature of 37 °C [66] and a replication-deficient self-erasable Sendai virus vector responding to miRNA 302, expressed by pluripotent cells [67], were developed to counter this drawback.

Consequently, the Sendai virus approach is now considered the gold standard method for cell reprogramming in research settings, and it may even be transferrable to clinics.

Episomal vectors

Episomes, which include plasmids and DNA minicircles, are extrachromosomal DNA molecules that can replicate autonomously in cells and can be used to transfect reprogramming factors directly and transiently into somatic cells.

Polycistronic plasmids or a pair of plasmids expressing two each, i.e., OCT4/SOX2 and KLF4/C-MYC, can be transfected to expressed OSKM. Although attractive for its simplicity, this technique requires repeated transfections, because plasmids only permit a time-restricted expression due to their becoming progressively lost in each cycle of cell division. To overcome this problem, Yu et al. used three oriP/Epstein–Barr nuclear antigen-1-based episomal vectors expressing different reprogramming factors and which can be removed from cells in the absence of drug selection [68]. However, the efficiency of this method remains very low, and only a third of the iPSCs are devoid of a DNA vector. Okita et al. improved this efficiency by using three episomal plasmid vectors, with five reprogramming factors OSKML and an additional shRNA against *TP53*, to reprogram human dermal fibroblast lines and two dental pulp cell lines [69]. However, there are safety concerns with *TP53* knockout, as it has been found to cause genomic instability [40].

DNA minicircles are circular episomal DNA vectors containing only the eukaryotic promoter and cDNAs of interest. Using this strategy, Jia et al. [19] and Narsinh et al. [20] reprogrammed human adipose stem cells into iPSCs with the OSKM combination. The reprogramming efficiency obtained is higher than with plasmids.

Protein delivery

The use of recombinant proteins as a method of delivering reprogramming factors is another way of not integrating foreign genetic material into the host cells. The first successful reprogramming mediated by recombinant proteins took place in 2009 on murine fibroblasts by Zhou et al. [70]. A poly-arginine (11R) protein transduction domain fused to the C-terminus of the four OSKM factors was used to facilitate protein entry into the cell. Adding valproic acid was then required to obtain iPSCs. The same year, Kim et al. reprogrammed human fibroblasts using extracts from HEK293 cell lines, each expressing one of the four OSKM factors [71]. More recently, other studies have demonstrated the reprogramming of somatic cells into iPSCs aided by, for example, reversible permeabilization mediated by streptolysin O [72] or cationic bolaamphiphiles [73].

The main advantage of using proteins is the safety of the method, as no integration into the genome is possible, but its low efficiency makes it less attractive for regenerative medicine.

Chemical molecules

Small chemical molecules not only enhance reprogramming but can also reprogram somatic cells alone. iPSCs have indeed been generated from murine somatic cells directly with a cocktail of chemical molecules by Hou et al. and Ye et al. [74,75]. Mouse embryonic fibroblasts (MEFs), neural stem cells (NSTs) and small intestinal epithelial cells (IECs) were thus reprogrammed by using the same core chemical cocktail and only fine-tuning the concentrations of the chemical compounds. Another study from Long et al. showed that bromodeoxyuridine (BrdU) can replace *Oct4*, and it was used with several chemical compounds to generate iPSCs from mouse fibroblasts [76]. Although the search for these small molecules is attractive for the generation of safe iPSCs for clinical applications, the reprogramming efficiency of this strategy remains low, and the use of chemical compounds to reprogram human somatic cells has not been reported to date.

RNA delivery

The emergence of RNA therapeutics as an alternative strategy is based on two natural advantages of synthetic RNAs: they do not integrate into the genome, and their delivery to the nucleus is not required. When applied to reprogramming, the direct delivery of synthetic mRNA into somatic cells to induce pluripotency is the most footprint-free and genomic integration-free strategy to generate iPSCs. Furthermore, RNA delivery has the highest reprogramming efficiency when compared with other nonviral nonintegrative delivery systems. To date, RNA delivery seems to be the only strategy to combine safety and efficiency features, and it is thus the most promising route for future clinical applications.

## 3. RNA-Based Reprogramming

### 3.1. RNA Technologies

#### 3.1.1. Synthetic RNAs

Synthetic mRNA production and modifications

**Figure 2:**
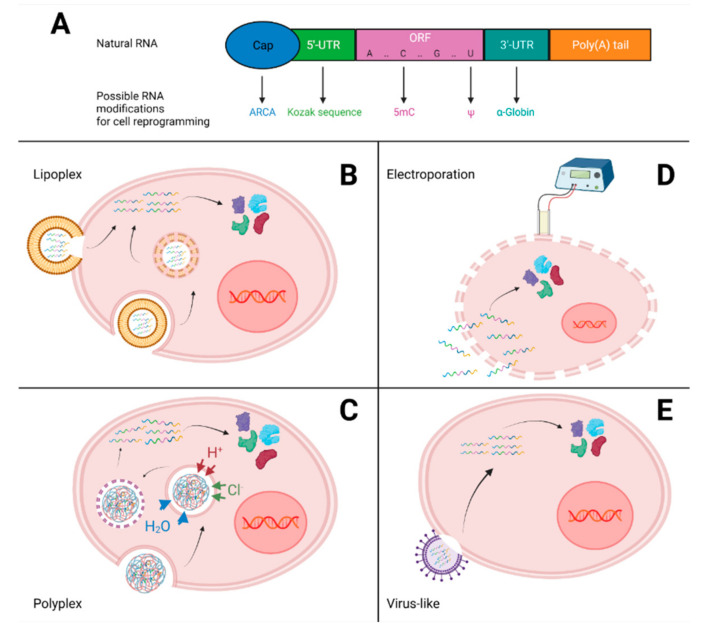
(**A**) The structure of a natural mRNA includes: a cap structure, untranslated regions (5′-UTR and 3′-UTR), an open reading frame (ORF) and a polyadenylated tail. Possible chemical modifications that are used in the field of cell reprogramming allow increasing the stability of mRNAs to reduce immunogenicity and to increase the transfection efficiency. The different RNA delivery methods developed here are nonviral and allow the introduction of mRNAs into the cytoplasm for translation into proteins without the need to integrate foreign genetic material into the host cell. (**B**) Lipoplexes can be integrated into the host cell by direct fusion with the membrane or by endocytosis, followed by destabilization of the endosomal membrane. (**C**) Polyplexes enter the cell by endocytosis and then release mRNAs into the cytoplasm by a “proton sponge” effect. (**D**) With electroporation, the cell is subjected to a rapid high-voltage current that causes a temporary permeability of the membrane, and the mRNAs can then enter the cell through these pores. (**E**) Virus-like particles allow the introduction of mRNAs into the host cell in a very specific and efficient way, using the properties of viruses but without having any integration or genomic trace. Created with BioRender.com.

As a string of nucleotides, RNA needs certain features to be functional in a cell. A mature messenger RNA is naturally composed of five domains: a cap structure at the 5′ end, a 5′-untranslated region (5′-UTR), an open reading frame (ORF), which is the coding sequence of the gene of interest, a 3′-untranslated region (3′-UTR) and a polyadenylated tail (poly-A tail) (Figure 2A). First of all, the 5′-cap consists of a methylated guanosine at position N7, linked to the first nucleotide by a 5′-5′-triphosphate (m^7^G). The cap impacts the mRNA stability and lifespan by blocking the action of exonucleases, but it also plays a role in the regulation of the export of mRNA from the nucleus. Once in the cytoplasm, the cap allows the translation of mRNA into a protein by binding to the initiation factor eIF4E [77]. In addition, the 5′-cap can also prevent mRNA recognition by the immune system [78,79]. Similarly, the poly-A tail protects mRNA from degradation by exonucleases, contributes to the export of mRNA from the nucleus to the cytoplasm and is essential for ribosome recruitment through its interaction with the poly(A)-binding protein (PABD) [80]. The 5′ and 3′ UTR both play a role in translation and mRNA stability, since the 5′-UTR interacts with the translation machinery [81], and the 3′-UTR serves as a binding site for miRNAs [82].

The latest advances in in vitro transcription methods have allowed the incorporation of chemical modifications to improve mRNA performance. Indeed, the instability and immunogenicity of mRNAs has hindered their use in therapeutic applications. The transfection of mRNAs induces an innate immune response through pattern recognition receptors (PRRs), including the activation of Toll-like receptors (TLR3, TLR7 and TLR8) and RNA sensors (retinoic acid-inducible gene-I like receptors), which induce a type I interferon (IFN)-mediated viral immune response [83,84,85,86,87,88,89]. The addition of these chemical modifications has created a new class of functional mRNAs: modified synthetic mRNAs. The following section aims to briefly describe the in vitro transcription methods and the main possible chemical modifications used for cellular reprogramming.

Regarding the 5′ cap, two methods can be used. The first one uses the Vaccina virus-derived capping enzyme to insert a natural unmodified cap at the 5′ end of the mRNA. This method allows a very high percentage of capping [90]. In the second method, a synthetic capping analog is synthesized together with the mRNA by adding a capping dinucleotide in the form of m7GpppG. The advantage of the second method is that a wide range of modified caps can be obtained. However, this method has a lower percentage of capping, and the capped dinucleotide can be inserted in both directions, leading, in one case, to a structure not recognized as a cap. To overcome this issue, modified cap dinucleotides have been synthesized so that they can only be inserted in the forward orientation [91]. This construction is called anti-reverse cap analog (ARCA) and increases the translation efficiency and stability of modified mRNAs [91,92].

The poly(A) tail can be added to synthetic mRNAs in two ways: either by being directly transcribed from the template vector or by being added to the mRNA post-transcriptionally in an enzymatic manner [93]. The limitation of the latter method is that it does not generate RNAs with poly(A) tails of equal lengths. Transcription, on the other hand, allows to obtain defined and homogeneous poly(A) tail lengths.

For the 3′- and 5′-untranslated regions, this strategy is often to use stable UTRs, such as those derived from α/β-globin genes, to allow for increased stability and translation efficiency [94].

Finally, the last possible modification involves substitution of the nucleotide with its modified equivalent throughout all the coding and noncoding sequences. It has been shown that several natural chemical modifications of nucleotides can reduce the innate immune response triggered by the recognition of an exogenous mRNA [95,96,97,98,99,100]. Cell death and toxicity caused by synthetic RNAs are thus reduced with these modifications. Among the most commonly used are 5-methyluridine (5mU), 5-methylcytidine (5mC), pseudouridine (ψ), N6-methyladenosine (6mA), N1-methylpseudouridine (1mψU) and 5-methoxyuridine (5moU) [101,102].

Reprogramming with synthetic mRNAs

The first somatic cell reprogramming based on modified synthetic mRNAs was reported by Warren et al. in 2010 [103]. Modified mRNAs encoding the five OSKML factors, in combination with valproic acid, were used to generate iPSCs from fetal fibroblasts, BJ postnatal fibroblasts and fibroblast-like cells cultured from a primary skin biopsy taken from an adult cystic fibrosis patient. Modifications to these mRNAs included: an anti-reverse cap analog, poly(A) tail, 5′ UTR containing Kozak sequence, α-globin 3′ UTR and complete substitutions of 5mC for cytidine and ψ for uridine. These modifications allowed repeated daily transfections without compromising the cell viability in contrast to unmodified RNAs. The cell viability was increased by supplementing the B18R inhibitor of interferon-mediated antiviral activity. In 2012, Warren et al. went on to use the same modifications to reprogram human fibroblasts, this time using a cocktail of six factors, including a variant of *OCT4* with a *MYOD* transactivation domain (M_3_O,SKMLN), allowing for a faster and more efficient induction of pluripotency [104]. M_3_O was indeed shown to effectively facilitate the chromatin remodeling of pluripotency genes [105,106]. In 2018, this same cocktail of six factors, with the same modifications, was used in combination with the miR-302-367 cluster to generate iPSCs from human fibroblasts of different ages, both healthy and diseased [107]. In 2020, that protocol was modified by McGrath et al. to reprogram the primary fibroblast lines associated with diseases or fibroblasts that were hitherto difficult to reprogram [108]. Additionally, with these same modifications, human newborn foreskin fibroblasts and skin fibroblasts from a patient with low-density lipoprotein receptor deficiency were reprogrammed into iPSCs with five OSKML factors in 2014 by Sjogren et al. [109] and in 2015 by Ramakrishnan et al., respectively [110].

With slightly different modified synthetic RNAs from all these studies, i.e., without α-globin in the 3′ UTR region but with an analogous anti-reverse cap analog, poly-A tail, 5′ UTR containing Kozak sequence, 3′ UTR and full substitution of 5mC for cytidine and ψ for uridine, Preskey et al. reprogrammed human BJ fibroblasts with a cocktail of six OSKMNL factors [111].

Mandal et al. suggested a protocol to reprogram primary human fibroblasts with modified RNAs (anti-reverse cap analog, poly(A) tail, 5′ UTR, 3′ UTR and full substitution with 5mC and ψ) encoding a cocktail of five OSKML factors [112].

Stemming from these pioneer studies, many reports have been published confirming the suitability of modified synthetic mRNA approaches for reprogramming. However, unmodified synthetic mRNAs have also been used to successfully generate iPSCs from different somatic cell types. One of the first protocols using unmodified mRNAs reported the generation of iPSCs differentiating into cells from the three embryonic layers in vivo by co-transfecting synthetic unmodified mRNAs encoding OSKMLN and immune-evading mRNAs encoding Vaccina Virus “E3 and K3 PKR inhibitors and B18R protein” (EKB). The addition of polycistronic miRNA cluster miR-302/367 allowed Poleganov et al. to obtain a robust protocol for the generation of iPSCs from human fibroblasts in 11 days with only four transfections or from human blood-derived endothelial progenitor cells in 10 days with eight transfections [113].

This protocol was, in fact, taken over by the Stemgen/Reprocell Company to commercialize a StemRNA™ 3rd Gen Reprogramming Kit with unmodified RNAs, which many research teams have used since to generate iPSC lines [114,115,116,117].

The major studies using modified and nonmodified synthetic mRNAs to reprogram human somatic cells are summarized in Table 1, along with the mRNA features, the reprogramming factors used and the starting cell types.

#### 3.1.2. Self-Replicative RNAs

The main disadvantage of synthetic RNAs is transient expression and the need for multiple transfections. To counter this, in 2013, Yoshioka et al. proposed the use of a self-replicative RNA (srRNA) to reprogram newborn fibroblasts or human dermal fibroblasts with a single transfection [129]. Their objective was to design an approach using an RNA species able to self-replicate over a limited number of cell divisions, to code for at least four reprogramming factors and to express these factors consistently at a high level and, finally, to be degraded in a controlled manner. To achieve this aim, they used a modified Venezuelan equine encephalitis virus (EEV) RNA replicon. This single-stranded RNA contains a sequence at the 5′ end that encodes four nonstructural proteins of the replication complex (nsP1–nsP4) separated from the coding sequences for the viral structural proteins at the 3′ end. In this first study, the authors replaced the coding sequences for the viral structural proteins by the coding sequences of *OCT4*, *KLF4* and *SOX2* separated by internal ribosomal skipping 2A peptides followed by an IRES and then *c-MYC* or *GLIS1*, a second IRES and a puromycin resistance gene [129]. This last gene allows a positive selection of the actually transfected cells, thanks to the addition of puromycin from days 2 to 10. Moreover, as the exposure of cells to this VEE RNA induces a strong interferon (IFN)-α/β innate immune response, the continuous exposure of cells to the B18R protein is essential for the preservation of the VEE RNA replicon and the generation of iPSCs. The removal of B18R induces selective degradation of the replicon. Thus, in the protocol, B18R is only added during the generation of iPSC colonies, and by passage 8, all iPSC clones have lost the replicon. This simple and straightforward new approach allowed iPSCs to be generated from newborn and adult human fibroblasts by a single transfection of a synthetic, polycistronic, self-replicative RNA replicon. It thus overcomes the two disadvantages associated with inefficient synthetic RNA reprogramming, since it requires only one transfection and expresses the four reprogramming factors at constant levels and ratios within a single cell.

Despite these improvements, the reprogramming efficiency of adult human dermal fibroblasts (>50 years) was very low. To improve the efficiency, in 2017, the same team designed a synthetic self-replicating srRNA with five factors (OCT4, KLF4, SOX2, GLIS1 and C-MYC) [130]. The construction of the srRNA was the same as in the previous study but with C-MYC added downstream of GLIS1 and both separated by a 2A peptide. In addition, adding a phosphatase treatment of srRNA, plus a new transfection reagent, also contributed to the significant improvement in transfection efficiency, avoiding puromycin selection. Thus, this novel five-factor synthetic self-replicative RNA was able to generate iPSCs from the fibroblasts of six adults aged 24–77 years, with a 4–10-fold increase in efficiency over the previous four-factor srRNA. The reprogramming kinetics were also improved by one week.

Other cell types have also been reprogrammed, with constructs very similar to the Yoshioka four-factor srRNA. For example, Umrath et al. used an srRNA encoding the four OSKM factors, and green fluorescent protein (GFP) containing an ORF for puromycin resistance, to reprogram human jaw periosteal cells in feeder- and xeno-free conditions [131]. In 2019, the same team used this srRNA construct to generate iPSCs from urine-derived renal epithelial cells [132]. Commercial kits have also been developed using this reprogramming technique. For example, the ReproRNA™-OKSGM kit (Catalog #05930) was recently launched by STEMCELL Technologies and used to reprogram urine-derived cells [133] or human olfactory neurosphere-derived cells [134]. Similarly, the StemRNA™-SR Reprogramming Kit from the Stemgent/Reprocell Company has been used to generate iPSCs from endothelial progenitor cells derived from umbilical cord blood or adult peripheral blood [135,136,137].

Finally, a study by Steinle et al. compared the efficiency of self-replicating RNA-encoding OSKM factors (and GFP) with synthetically modified messenger RNAs encoding the five OSKML reprogramming factors. Although both methods generate iPSCs without integration and without genomic alteration, the authors concluded that srRNA-based reprogramming is more efficient and practical than mRNA-based reprogramming. They reported a lower cost, time-savings with a single transfection, a higher efficiency with the positive selection by puromycin and, finally, direct control of the transfection thanks to GFP [138].

#### 3.1.3. MicroRNAs

MicroRNAs are small, single-stranded, noncoding RNAs of about 22 nucleotides that control gene expression at the post-transcriptional level. Indeed, they are able to turn off the expression of a gene by binding to the sequences of messenger RNAs to degrade them or inhibit their translation. Very early on, it was reported that miRNAs play a crucial role in the cellular reprogramming towards pluripotency.

The first proof that somatic cells can be reprogrammed purely by the expression of miRNAs was provided in 2008 by Lin et al. [139]. They demonstrated that the expression of the miR-302-367 cluster with a retroviral Pol-II-based intronic miRNA expression system can reprogram human cancer cells into ESC-like pluripotent stem cells. Later, the same result was obtained with normal human hair follicle cells and a novel inducible pTet-On-tTS-miR302 expression vector in conjunction with electroporation delivery [140]. Anokye-Danso et al. also used the miR-302-367 cluster but with a lentivirus delivery system to reprogram human dermal and foreskin fibroblasts, with a reprogramming efficiency higher than that obtained with the OSKM factors, reaching 10% [141]. The miR-302-367 cluster is composed of five miRNAs: miR-302a, miR-302b, miR-302c, miR-302d and miR-367, which target over 445 human genes. Although its precise mode of action is difficult to establish, the miR-302-367 cluster could reprogram somatic cells through three main pathways: (i) by targeting various epigenetic factors (AOF2, DNMT1 and MECP1/2) allowing the global demethylation of genomic DNA and modifications of H3K4 [140]; (ii) by strongly interacting with Oct4/Sox2 [142] and (iii) by facilitating mesenchymal–epithelial transition (MET) [143,144,145]. Moreover, repression of the miR-302-367 cluster has been shown to dramatically alter the reprogramming of human foreskin fibroblasts [146].

Another team successfully reprogrammed human dermis fibroblasts and human adipose stromal cells using only miRNAs [147]. In contrast to the studies mentioned above, they used miR-200c expression in combination with miRNAs from the miR-302-367 and miR-369 clusters without retroviral or lentiviral vectors. They performed direct transfections of mature miRNAs, resulting in integration-free iPSCs. Unfortunately, several teams have failed to reproduce this result. Hu et al. were unable to reprogram human adipose-derived stem cells with a lentivirus delivery system [145], Lu et al. failed to reprogram mouse embryonic fibroblasts using a PiggyBac transposon to carry the miRNAs [148] and Lee et al. failed to reprogram normal human fetal lung fibroblasts and primary CD34^+^ cells derived from cord blood transduced with lentiviruses overexpressing the miR-302-367 cluster or with the miR-302 cluster alone [149]. These differences can be explained by the miRNAs used, the delivery techniques employed or the starting cell types.

Despite these teething problems, the influence of miRNAs on the reprogramming efficiency of somatic cells has been observed many times. miRNAs known to improve cell reprogramming include miRNAs expressed by pluripotent cells or, on the contrary, miRNAs acting as a barrier, often miRNAs specific to the starting cell type.

Among the miRNAs known to enhance cell reprogramming, the miR-302-367 cluster as a whole or its members separately have been reported many times. Thus, studies that failed to reprogram somatic cells with these miRNAs alone were still observed to have an increase in reprogramming efficiency [145,148,149]. The miRNAs that have been reported to enhance cellular reprogramming in human cells are listed in Table 2. miRNAs known to inhibit reprogramming in humans include miR-145 [150] and miR-29a [151].

Using miRNAs for somatic cell reprogramming has several advantages. First, because of their small size, miRNAs are easier to transfect than mRNAs or other reprogramming vectors. Moreover, the use of miRNAs during reprogramming obviates the need for the C-MYC oncogene, making the generation of iPSCs safer. Finally, their role as reprogramming enhancers allows miRNAs to increase the reprogramming efficiency and/or to decrease the number of transfections required when used in conjunction with mRNAs.

#### 3.1.4. CRISPR-Cas9

Although not a totally RNA-based method, CRISPR approaches deserve some attention here. Original methods recently emerged for cell reprogramming based on the CRISPR system and, more precisely, the CRISPR activation system (CRISPRa). It is based on the expression of a catalytically inactivated form of Cas9, dead Cas9 (dCas9), fused with transcriptional activation domains. When directed to DNA regions using synthetic single guide RNAs (sgRNA), this increases the expression of endogenous genes of interest. Thus, the use of this technology with all its derivatives is of great interest for the fields of cell reprogramming and regenerative medicine [156,157].

In 2018, two studies reported the generation of iPSCs from somatic cells using only CRISPRa. First, Liu et al. used CRISPRa to target a single *Sox2* locus or the *Oct4* promoter and enhancer simultaneously in mouse embryonic fibroblasts [158]. In both cases, activation of these loci resulted in the induction of other pluripotency genes and thus generated iPSC lines. Later, Welter et al. were the first to report the generation of iPSCs from human somatic cells using only CRISPRa [159]. Initially they reprogrammed neuroepithelial stem cells (NSCs) by activating endogenous OCT4 just with CRISPRa. Then, they generated iPSCs from primary human skin fibroblasts by targeting endogenous OCT4, SOX2, KLF4, MYC and LIN28A promoters. Since the reprogramming efficiency was low, they also targeted the sequence EGA-enriched Alu-motif (EEA-motif), which is likely to be involved in the control of early embryonic transcriptional networks, to significantly increase the generation of iPSCs [159].

These two inspiring publications proved that, although the use of CRISPR technology for the production of pluripotent cells is still in its infancy, this approach is very promising.

### 3.2. RNA Delivery

Once the mRNAs encoding the pluripotency transcription factors have been selected and transcribed, a key element for efficient and safe reprogramming is the delivery strategy. Indeed, it is necessary to transport the mRNAs inside the cytoplasm of the target cells. However, the first obstacle to transfection is that naked mRNAs can be degraded by a gamut of nucleases present in the serum and in the cell or by immune cells, even if the mRNAs are chemically modified. Moreover, due to their negative charge, hydrophilic nature and high molecular weight, mRNAs alone are unable to pass through the cell membrane, which contains negatively charged phospholipids.

Due to their limitations for mRNA delivery and the safety concerns that arise, the viral vectors will not be presented here. Instead, we will detail the most widely used nonviral physical and chemical methods in the field of RNA-based reprogramming.

#### 3.2.1. Lipoplex

Cationic lipids are the most common chemical carriers for RNA delivery in reprogramming. They consist of a positively charged hydrophilic polar head, one or two lipophilic hydrocarbon chains and a linking group. The polar head allows an electrostatic interaction with the negatively charged RNA, while the lipophilic carbon chains are involved in the supramolecular assembly of the complex and will facilitate interactions with the cell membrane. The cationic RNA–lipid complex forms a continuous spherical bilayer structure, containing the RNAs in the center, called a lipoplex. The lipoplex is then integrated into the host cell by endocytosis or membrane fusion, and the transfected cells rapidly express the gene of interest (Figure 2B). This transfection with cationic lipids is called lipofection. The efficiency of lipofection depends on the ability of the lipid to complex with and then release the RNAs. Neutral “helper lipids” can also be added to improve the stability, half-life and, thus, the transfection efficiency of the lipoplex.

Several commercial transfection reagents were developed and widely used for RNA-based reprogramming. Among the most widespread, Lipofectamine^®^ RNAiMAX [103,104,107,108,112,121,122,127,160], Lipofectamine^®^ 2000 [118], Stemfect™ [124] and FuGENE^®^ [120] can be mentioned.

#### 3.2.2. Polyplex

Polymeric cations can also be used as an RNA delivery strategy. Cationic polymers, with an n-fold repeating chemical unit, carry multiple positive charges. They allow the complexation of RNA through electrostatic interactions between cationic groups of the polymer and the negatively charged nucleic acids. This kind of structure is called a polyplex. The most explored polymer for nucleic acid delivery is polyethyleneimine (PEI). Its high charge density binds strongly to nucleic acid, causing uptake by the cells and the excellent intracellular release of RNA from endosomes based on the “proton sponge” effect [161] (Figure 2C). Unfortunately, PEI is also somewhat toxic to cells.

Recently, Choi et al. used a functionalized PEI with graphene oxide to complex and release mRNAs encoding OSKM in human adipose tissue-derived fibroblasts [126]. They demonstrated that the combination of negatively charged and cationic PEI decreased the cytotoxicity while successfully complexing the RNA. Furthermore, graphene oxide–PEI complexes effectively protect mRNAs from RNase A, and their transfection into cells in dynamic suspension significantly increases the transfection efficiency, allowing daily transfections to be avoided. Thus, with this vector, iPSCs can be generated with just three transfections at 48 h apart.

#### 3.2.3. Electroporation

Physical methods allow the direct transfection of RNAs into the cell, and the most widespread is electroporation. It is based on the principle of creating a temporary permeability of cell membranes following exposure to a rapid high-voltage current. RNAs can then enter into the cytoplasm through these pores (Figure 2D).

In 2010, Plews et al. developed an electroporation-based protocol to transfect human fibroblasts efficiently with modified mRNAs encoding *OCT4*, *SOX2*, *KLF4*, *C-MYC* and SV40 large T [119]. They showed that, after a single transfection, the cells expressed these factors at levels similar to, or higher than, human ESCs and that the addition of small molecules (5-aza-29-deoxycytidine, BIX-01294 and valproic acid) resulted in small aggregates with alkaline phosphatase activity and OCT4 protein expression. However, these aggregates grew very slowly and could not be passaged. Unfortunately, electroporation caused a high rate of cell death and senescence.

To overcome this issue, Arnold et al. used a transfection protocol for different combinations of reprogramming factors with a single electroporation followed by three lipofections every 72 h [120]. As before, electroporation itself was efficient at transfection, but it resulted in a high cell toxicity. Nevertheless, they were able to generate Huntington-specific iPS cell lines through this nonintegrating reprogramming method. Another way to circumvent the cellular toxicity of electroporation is to use a self-replicative RNA, since it requires only a single transfection. In 2020, Bouma et al. published a protocol for reprogramming urine-derived cells using a commercial self-replicative RNA kit and a single electroporation [133].

#### 3.2.4. Virus-Like Particles

Other RNA delivery strategies have been developed based on what could be learned from viruses. Such virus-like particles allow transferring RNAs into cells of interest in a very specific and efficient manner, allowing for broad clinical applications like gene editing or regenerative medicine, and might be tremendous tools for cell reprogramming in the future (Figure 2E).

A first approach developed by Prel et al. took advantage of an RNA delivery system based on a bacteriophage–lentivirus chimera [162]. It has been constructed by exploiting a bacteriophage coat protein and its cognate 19-nt stem loop, instead of the natural lentiviral Psi packaging sequence, to achieve mRNA packaging into the lentiviral vectors. This strategy allows a safe and efficient nonviral RNA delivery in vitro and in vivo. This transient RNA expression could be achieved without retro-transcription or integration or any genomic trace. Moreover, one of the exciting features of these virus-like particles is the possibility of selecting the viral envelope exposed at their surfaces to target specific cells or enhance the transduction efficiency.

Using similar principles, Segel et al. developed a so-called “selective endogenous encapsidation for cellular delivery” (SEND) approach to package, secrete and deliver specific RNAs [163]. They identified a retroviral-like protein, PEG10, which directly binds to mRNA and can assemble in virus-like capsids. The authors also added the fusogen vesicular stomatitis virus envelope to the particles to facilitate cellular delivery. Thus, here again, the flexibility associated with pseudotyping could greatly increase the versatility of such virus-like particles to deliver functional mRNA cargos into mammalian cells.

Clearly, these new methods offer particularly attractive prospects to significantly increase the potential applications of RNA-based technologies for reprogramming. Beyond their efficiency and specificity, these techniques also open up the possibility of precisely, quantitatively, temporally and spatially controlling the expressions of the genes of interest.

## 4. Transient Reprogramming

Although it is intuitive that reprogramming might promotes cell rejuvenation, as an embryonic cell (or iPSC) has more juvenile features than an adult cell, the forced and maintained expression of OSKM was repeatedly described to favor cellular senescence [39,40,41,42,43,44]. To overcome this barrier, we demonstrated, for the first time, that cell reprogramming can even rejuvenate cells from centenarians and that it can also overcome the barrier of cell senescence without directly inactivating senescence inducers such as p53, p16INK4A and p21CIP1 [38]. The reprogramming protocol used was based on the use of a cocktail of the combined six reprogramming factors from pooling the overlapping four factor cocktails of Yamanaka [5] and Thomson [7], i.e., OCT4, SOX2, KLF4, C-MYC, NANOG and LIN28 (OSKMNL). Following this protocol, we discovered that iPSCs reprogrammed from replicative senescing or centennial cells had restored the telomere and mitochondrial functions with a gene expression profile similar to ESCs. In addition, after their redifferentiation, the fibroblasts obtained had reset their proliferation capacity and had a similar transcriptomic profile to the fibroblasts derived from hESCs, as well as a restored metabolism. This demonstrated conclusively that “cellular aging” is reversible [38]. Horvath proposed an epigenetic clock based on DNA methylation to estimate the biological age and demonstrated that iPSCs reset their epigenetic age to that of ESCs [164]. These research avenues all confirm that cellular age can be reversed.

These seminal results led the scientific community to ask whether cellular rejuvenation due to reprogramming could take place in vivo. To answer this question, Abad et al. were the first to generate transgenic mouse models, expressing OSKM under the control of doxycycline. Strikingly, they observed the emergence of teratomas in several organs, thus demonstrating the feasibility of in vivo reprogramming [165]. However, to prevent deterioration related to aging or to rejuvenate the organism, it is important not to generate fully dedifferentiated cells, as this leads to a deterioration of the animal’s health or tumor formation. Consequently, it was judicious to think to trigger the reprogramming process and stop it before obtaining pluripotent cells, hoping that it might erase cellular aging marks instead of favoring senescence. Ocampo et al. envisioned such a strategy and proposed a protocol to induce partial reprogramming in a homozygous progeria transgenic mouse model. They induced OSKM expression for 2 days per week during the lives of the animals with doxycycline and observed a significant increase in the lifespan of these animals, as well as the improvements in age-related hallmarks [166]. In addition, they showed that the induction of OSKM improves the regenerative capacities of the pancreas and muscles of non-progeria animals after injury. Other teams also demonstrated the benefits of partial reprogramming in vivo with cyclic protocols [167] or by short inductions [168,169,170]. In our laboratory, we discovered that a short transient cellular reprogramming with a punctual induction of the Yamanaka factors for only 2.5 weeks at the age of two months could have a distal impact either on the lifespan and health span of heterozygous progeria mice [170].

This partial or transient reprogramming, which consists of inducing the expression of reprogramming factors to initiate reprogramming without ever completing it, thus allowing the preservation of the cellular identity, has been further studied in vitro. Cellular reprogramming is indeed composed of three phases: initiation, maturation and stabilization, and it is only during the last phase that cells acquire transgene-independent self-renewal and pluripotency while losing their epigenetic memory [171]. Intriguingly, Olova et al. found that the loss of somatic gene expression and epigenetic age can be uncoupled, which implies the existence of a safe window where rejuvenation can be achieved with a minimized risk of cancer [172]. Studies have therefore focused on transiently initiating reprogramming up to the initiation or maturation phases.

In 2020, Sarkar et al. developed an in vitro transient reprogramming strategy within the initiation phase that uses mRNAs to induce six OSKMLN reprogramming factors for four days in the fibroblasts and endothelial cells [173]. By analyzing the transcriptomic signatures of young, aged or transiently reprogrammed cells of both cell types, they found that the expression of cell identity genes was not affected and that the transiently reprogrammed cells switched immediately to a younger gene expression profile. To further quantify this rejuvenation, a pan-tissue epigenetic clock and a skin-and-blood clock analysis of DNA methylation demonstrated the age reversal. The transient expression of OSKMLN also restored many of the cellular characteristics impaired with age in these two cell types. As such, the epigenetic repressive mark H3K9me3, the heterochromatin-associated protein HP1γ and the nuclear lamina support protein LAP2α were increased, more autophagosomes formed and the proteosomal activity was enhanced and the mitochondrial membrane potential was increased, while the mitochondrial oxygen radicals were decreased. Finally, among the endothelial cells, only the number of senescent cells was decreased, along with reduced proinflammatory cytokines of the senescence-associated secretory phenotype. To analyze whether the transient expression of OSKMNL could also reverse age-related inflammatory phenotypes, Sarkar and coworkers transfected OSKMLN mRNAs into chondrocytes from elderly patients with osteoarthritis for 2 or 3 days. This caused a significant reduction in the intracellular RANKL and iNOS2 mRNA levels and levels of the inflammatory factors secreted by the cells (MIP1A, IL-6, IFNA and MCP3). An increase in cell proliferation and ATP production, as well as a decrease in oxidative stress, were also observed, thus confirming the impact on inflammation [173]. Finally, transplanted into a mouse model of muscle injury, transiently reprogrammed murine and human muscle stem cells of different ages led to improvement in the tissue regeneration potential [173]. This seminal study demonstrates that nonintegrative transient cell reprogramming, fully exploiting the potential of RNAs, can rapidly reverse many hallmarks of aging in multiple cell types while retaining their identity, constituting a footprint-free approach much more easily translatable to the clinic.

A recent mouse model developed by Lu et al. demonstrated the potential of transient reprogramming as a strategy for rejuvenation and regeneration [174]. The group did not use a transgenic model, as in previous studies. Rather, they performed ectopic expression of the reprogramming factors via a dual adeno-associated virus containing a polycistronic cassette coding for OSK that they injected into the vitreous body of the mouse eye. A prolonged induction for more than 15 months was performed to ensure the safety of this system without any retinal deformation or increase in tumor incidence. No loss of cell identity or pluripotency was observed. The effects of OSK induction were then tested in three different models: an optic nerve crush injury model, glaucoma model induced by elevated intraocular pressure and vision loss caused by natural aging. They found an increase in the survival of retinal ganglion cells, regeneration of their axonal extension and the recovery or restoration of vision associated with a young gene expression and epigenetic signatures. Interestingly, inhibition of the DNA demethylases TET1 and TET2 prevents restoration, demonstrating that DNA methylation regulations are essential in these regeneration phenomena [174]. Although this study did not use a technology based on RNA, this work is highly demonstrative of what can be expected in terms of rejuvenation strategies and highlights the potential interventional value of RNA technology in the context of cellular reprogramming in a specific situation where it is necessary to have a targeted and transitory action.

## 5. Concluding Remarks

There have been enormous technical advances in RNA-based reprogramming over the last decade. We now have many options where judicious choosing, between mRNAs, self-replicative RNAs and miRNAs, allows us to adapt to the constraints of different situations according to the cell type, the number of transfections or the required efficiency. Moreover, the design and modifications of RNAs have allowed a greater stability, a lower immunogenicity and a higher reprogramming efficiency. Finally, the numerous nonviral delivery approaches protect RNAs until they are translated in the cell. These technologies have also further improved the transfection efficiency while guaranteeing the generation of safe iPSCs. The recent emergence of virus-like particles as a delivery strategy even enables in vivo cell- or tissue-specific targeting.

By combining the safety and efficiency features, RNA-based reprogramming has generated and continues to attract interest from research teams worldwide. It is indeed a particularly promising field for rejuvenation and regeneration, where a large number of challenges still remain to be addressed before translation into the clinic. Clearly, this technology has a promising future with wide clinical applications, and ongoing studies will allow the emergence of critical masses in regenerative biology hubs with sufficient combined expertise to contribute to the development of this cutting-edge health technology.

## Figures and Tables

**Table 1:** Major studies using mRNA-based cell reprogramming.

Transcription Factors	Transfection Regent	Starting Cell Type	mARN Features	References	Authors
OSNL	Lipofectamine 2000	Human foreskin fibroblasts	Anti-reverse cap analog (ARCA) IRES sequence Poly(A) tail	[118]	Yakubov
OSKM OSKML	RNAiMAX	Human fibroblasts	ARCA 5mC and ψ5′-UTR containing Kozak sequence α-Globin 3′-UTR Poly(A) tail	[103]	Warren
OSKMT	Electroporation	Human fibroblasts	5′ and 3′ UTRs of Xenopus β-globin Poly(A) tail	[119]	Plews
OSK OSN ONhT OMN OKN OSKMNhT	Electroporation and FuGENE HD	Human fibroblasts	Cap Poly(A) tail	[120]	Arnold
M_3_OSKM^a^L M_3_OSKM^a^LN	RNAiMAX or Stemfect	Human fibroblasts	ARCA 5mC and ψ 5′-UTR containing Kozak sequence α-Globin 3′-UTR Poly(A) tail	[104]	Warren
OSKML	RNAiMAX or Stemfect	Human fibroblasts	ARCA 5mC and ψ 5′-UTR 3′-UTR Poly(A) tail	[112]	Mandal
OSKML	RNAiMAX	Adipose tissue-derived mesenchymal stem cells	Synthetic modified mRNA (5mC and ψ) from Stemgent	[121]	Heng
OSKML	RNAiMAX	Human fibroblasts	ARCA 5mC and ψ	[122]	Durruthy-Durruthy
OSKML	RNAiMAX	Newborn foreskin fibroblasts	Stemgent mRNA Reprogramming Kit	[109]	Sjogren
OSKML	RNAiMAX	Bone marrow–derived mesenchymal stromal cells	Synthetic modified mRNA (5mC and ψ) from Stemgent	[123]	Varela
OSKMLN +EKB +miR302a–d +miR367	RNAiMAX	Human fibroblasts and blood-derived endothelial progenitor cells	ARCA 5′-UTR containing Kozak sequence 5mC and ψ or not α-Globin 3′-UTR 3′-human-β-globin-UTR Poly(A) tail	[113]	Poleganov
OSKML	RNAiMAX	Human fibroblasts	ARCA 5mC and ψ5 ′-UTR containing Kozak sequence α-Globin 3′-UTR Poly(A) tail	[110]	Ramakrishnan
OSKML +miR302a–d +miR367	Stemfect	Human adult dermal fibroblasts	Synthetic modified mRNA (5mC and ψ) from Stemgent	[124]	Lee
ONhT OSK OSKMNhT	jetPEI	Human fibroblatsts	Cap 5′-UTR containing Kozak sequence Poly(A) tail	[125]	Rohani
OSKMLN	Stemfect	Human fibroblasts	6F mRNA Reprogramming Premix – Allele Biotechnology	[111]	Preskey
Natural mRNA extracted from HEK 293T or OSKM	Graphene oxide-polyethylenimine (Graphene oxide -PEI)	Human adipose tissue-derived fibroblasts	Natural mRNA extracted from HEK 293T Or Cap 5′-UTR 3′UTR Poly(A) tail	[126]	Choi
OSKML	RNAiMAX	Human amniotic fluid-derived stem cells	TriLink Biotechnologies Inc	[127]	Velasquez-Mao
M_3_OSKMLN +miRNA-367/302s	RNAiMAX	Human fibroblasts	ARCA 5mC and ψ Poly(A) tail	[107]	Kogut
M_3_OSKMLN +miRNA-367/302s	RNAiMAX	Human fibroblasts	ARCA 5mC and ψ 5′-UTR containing Kozak sequence α-Globin 3′-UTR Poly(A) tail	[108]	McGrath
OSKMLN +EKB +miR from miR302/367 cluster	RNAiMAX	Human Mesenchymal Stromal/Stem Cells	StemRNA^TM^ 3rd Gen Reprogramming Kit	[128]	Jeriha

O = OCT4, S = SOX2, K = KLF4, M = C-MYC, L = LIN28, M_3_O = MYOD-OCT4 fusion constructs, M^a^ = C-MYC-T58A, N = NANOG, hT = hTERT and T = SV40 large T.

**Table 2:** Main miRNAs described to enhance cell reprogramming.

miRNAs	Starting Cell Types	Reprogramming Factors	Reference	Authors
miR-302 cluster (without miR-367)	Human adipose stem cells	OSKM	[145]	Hu
miR-302 cluster (without miR-367)	Human CD34+ cord blood cells	OSKM	[149]	Lee
miR-302-367 cluster	Human primary neonatal fibroblasts	OSKMLN	[107]	Kogut
miR-302-367 cluster	Human fibroblasts (CRL-2097)	OSK	[143]	Liao
miR-302b or/and miR-372	Human foreskin (BJs) or lung (MRC-5) fibroblasts	OSKM or OSK	[144]	Subramanyam
miR-17-92 cluster or only miR-19a and miR-19b	Human fibroblasts (IMR90)	OSKM or OSK	[152]	He
miR-524-5p	Human foreskin fibroblasts (HFF-1)	OSKM	[153]	Nguyen
miR-371 cluster	Human fibroblasts (IMR90)	OSK	[154]	Cao
miR-31	Human CD34+ cord blood cells	OSKM	[155]	Lee
